# Human Immunodeficiency Virus Impairs Reverse Cholesterol Transport from Macrophages

**DOI:** 10.1371/journal.pbio.0040365

**Published:** 2006-10-31

**Authors:** Zahedi Mujawar, Honor Rose, Matthew P Morrow, Tatiana Pushkarsky, Larisa Dubrovsky, Nigora Mukhamedova, Ying Fu, Anthony Dart, Jan M Orenstein, Yuri V Bobryshev, Michael Bukrinsky, Dmitri Sviridov

**Affiliations:** 1 The George Washington University, Washington, District of Columbia, United States of America; 2 Baker Heart Research Institute, Melbourne, Victoria, Australia; 3 University of New South Wales, Sydney, New South Wales, Australia; St. Vincent's Hospital, Australia

## Abstract

Several steps of HIV-1 replication critically depend on cholesterol. HIV infection is associated with profound changes in lipid and lipoprotein metabolism and an increased risk of coronary artery disease. Whereas numerous studies have investigated the role of anti-HIV drugs in lipodystrophy and dyslipidemia, the effects of HIV infection on cellular cholesterol metabolism remain uncharacterized. Here, we demonstrate that HIV-1 impairs ATP-binding cassette transporter A1 (ABCA1)-dependent cholesterol efflux from human macrophages, a condition previously shown to be highly atherogenic. In HIV-1–infected cells, this effect was mediated by Nef. Transfection of murine macrophages with *Nef* impaired cholesterol efflux from these cells. At least two mechanisms were found to be responsible for this phenomenon: first, HIV infection and transfection with *Nef* induced post-transcriptional down-regulation of ABCA1; and second, Nef caused redistribution of ABCA1 to the plasma membrane and inhibited internalization of apolipoprotein A-I. Binding of Nef to ABCA1 was required for down-regulation and redistribution of ABCA1. HIV-infected and *Nef*-transfected macrophages accumulated substantial amounts of lipids, thus resembling foam cells. The contribution of HIV-infected macrophages to the pathogenesis of atherosclerosis was supported by the presence of HIV-positive foam cells in atherosclerotic plaques of HIV-infected patients. Stimulation of cholesterol efflux from macrophages significantly reduced infectivity of the virions produced by these cells, and this effect correlated with a decreased amount of virion-associated cholesterol, suggesting that impairment of cholesterol efflux is essential to ensure proper cholesterol content in nascent HIV particles. These results reveal a previously unrecognized dysregulation of intracellular lipid metabolism in HIV-infected macrophages and identify Nef and ABCA1 as the key players responsible for this effect. Our findings have implications for pathogenesis of both HIV disease and atherosclerosis, because they reveal the role of cholesterol efflux impairment in HIV infectivity and suggest a possible mechanism by which HIV infection of macrophages may contribute to increased risk of atherosclerosis in HIV-infected patients.

## Abstract 2

HIV1-Nef impairs ABCA1-dependent cholesterol efflux from infected macrophages, promoting the transformation of virally infected macrophages into foam cells (a condition that may put HIV patients at risk for atherosclerosis).

## Introduction

Macrophage cells and cholesterol play a central role in the pathogenesis of two diseases, AIDS and atherosclerosis. Macrophages are among the main targets of HIV in the body, and HIV assembly and budding, as well as infection of new target cells, all depend on plasma membrane cholesterol. Depletion of cellular cholesterol markedly and specifically reduces HIV-1 particle production [1,2], and cholesterol-sequestering drugs, such as β-cyclodextrin, render the virus incompetent for cell entry [3,4]. Therefore, regulating cholesterol delivery to nascent virions would be highly beneficial for the virus. Some clues to potential mechanisms that may be employed by HIV to achieve this goal have been provided recently when it was demonstrated that HIV-1 accessory protein Nef binds cholesterol and may deliver it to the site of virion assembly at the plasma membrane [5]. However, little is known about the relation of this mechanism to major cellular cholesterol trafficking pathways and about the effect of HIV infection on lipid metabolism in the host cells.

Increased risk of atherosclerosis and coronary artery disease (CAD) is a recognized clinical problem in HIV-infected patients [6–10]. A key element of atherosclerotic plaque is formation of foam cells [11], the majority of which are macrophages overloaded with cholesterol. Foam cells undergo apoptosis, necrosis, and calcification, and cholesterol released from foam cells forms the lipid-rich core of the plaque. Interestingly, foam cells are less conducive than macrophages to growth of certain pathogens, such as Chlamydia pneumoniae [12], suggesting that they may contribute to antibacterial response of the organism. One of the main causes of foam cell formation and accumulation of cholesterol in the vessel wall may be dyslipidemia, especially high levels of very low-density lipoprotein (VLDL) and low-density lipoprotein (LDL), as well as low levels of high-density lipoprotein (HDL) [13], and/or impairment of intracellular cholesterol metabolism. Dyslipidemia caused by antiretroviral drugs, in particular protease inhibitors (PIs) [14], is a known risk factor in pathogenesis of CAD in HIV-infected patients. One mechanism of PI treatment–dependent dyslipidemia is inhibition of degradation of proteins involved in metabolism of cholesterol and triglycerides, such as apolipoprotein B [15] or the adipocyte determination- and differentiation-dependent factor 1/sterol regulatory element binding protein 1 [16]. In addition to causing dyslipidemia, PIs may contribute to initiation of atherosclerosis by affecting intracellular metabolism of cholesterol via up-regulation of the scavenger receptor CD36 and the subsequent accumulation of sterol in macrophages [17]. Therefore, PIs affect both extra- and intracellular pathways of cholesterol metabolism and are likely an important factor in pathogenesis of atherosclerosis in HIV-infected patients. However, a number of clinical reports found a correlation between heart disease and HIV viral load, and detected an increased risk of CAD in patients not treated with PIs or even in drug-naive HIV-infected individuals [18,19]. Recent results support the role of HIV infection as an independent risk factor of CAD [7,9,10,20–22], but the mechanisms of this atherogenic effect of HIV remain unknown.

In this report, we identify one such mechanism. We show that HIV, via its accessory protein Nef, affects normal function of ATP-binding cassette transporter A1 (ABCA1) and consequently impairs cholesterol efflux from infected macrophages. Previous studies demonstrated the key role of ABCA1 in reverse cholesterol transport and found that functional mutations in ABCA1 cause Tangier disease, which is characterized by severe HDL deficiency, accumulation of sterols in tissue macrophages, and accelerated atherosclerosis [23]. The effect of HIV-1 on ABCA1 is consistent with ABCA1 inhibition described for viral [24] and bacterial [25] infections; however, the mechanism of impairment is unique for HIV. As a result of ABCA1 inhibition, HIV-infected macrophages accumulate lipids and transform into foam cells, a step that may contribute to the increased risk of atherosclerotic plaque formation. Our results also suggest that cholesterol efflux impairment may be a mechanism that ensures access of nascent virions to intracellular cholesterol, which is critical for HIV infectivity. Therefore, pharmacological stimulation of cholesterol efflux may be considered as a novel anti-HIV therapeutic strategy.

## Results

### Impairment of Cholesterol Efflux in HIV-Infected Macrophages

Cholesterol efflux is a pathway for removing excessive cholesterol from cells to extracellular acceptors. It is the first step of reverse cholesterol transport, and it plays a key role in maintaining cell cholesterol homeostasis. Impairment of cholesterol efflux leads to accumulation of intracellular cholesterol [26] and development of atherosclerosis in animal models [27] and in humans [28,29]. Analysis of cholesterol efflux from monocyte-derived macrophages infected in vitro with HIV-1 ADA [30] demonstrated a substantial inhibition of apolipoprotein A-I (apoA-I)-specific efflux (Figure 1A). A similar effect was observed in macrophages infected with two primary macrophage-tropic HIV-1 strains, Yu-2 and 92US660, indicating that impairment of cholesterol efflux is a general feature of HIV-1 replication in macrophages (Figure 1A). The level of cholesterol efflux inhibition correlated with the level of virus replication (Figure 1A). Importantly, at the time of analysis (21 d after infection), 80%–90% of the cells infected with ADA and Yu-2 viruses were p24^+^ (only 20% of the cells infected with 92US660 strain were p24^+^ at that time), indicating that reverse transcription (RT) values in these infections reflected the amount of virus produced per cell and that cholesterol efflux impairment depended on the level of virus protein expression.

**Figure 1:**
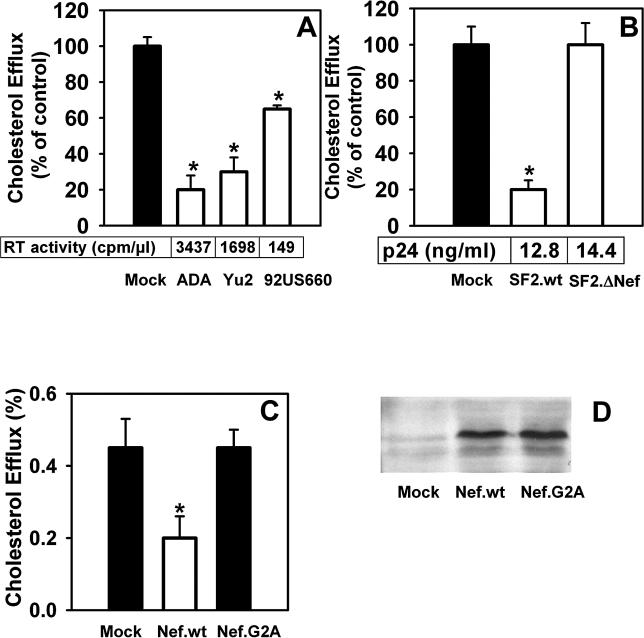
HIV-1 Nef Impairs Cholesterol Efflux from Macrophages (A) Monocyte-derived macrophages were inoculated with indicated HIV-1 strains equalized according to RT activity and cultivated for 21 d (RT activity in the culture supernatants on day 21 is shown beneath the bars). Mock-infected cells were incubated with virus-free medium. Specific cholesterol efflux to apoA-I (30 μg/ml) was performed for 12 h and analyzed as described in Materials and Methods. Results are presented as percentage of efflux from mock-infected cells (taken as 100%) and are mean ± standard deviation (SD) of triplicate determinations. An asterisk (*) indicates *p* < 0.001 (B) Monocyte-derived macrophages were inoculated with VSV-G-pseudotyped HIV-1 SF2 either deficient in Nef (SF2.ΔNef) or carrying WT Nef (SF2.wt). Specific cholesterol efflux to apoA-I (30 μg/ml) was analyzed on day 6 after inoculation using the same procedure as described in (A). p24 concentration in the culture medium is shown beneath the bars. Results are presented as percentage of efflux from mock-infected cells (taken as 100%) and are mean ± SD of triplicate determinations. An asterisk (*) indicates *p* < 0.001. (C) RAW 264.7 cells were transfected with plasmids expressing indicated Nef variants or an empty vector (mock-transfection). Twenty-four hours after transfection, LXR agonist, TO-901317 (1 μmol/L), was added. Cholesterol efflux to apoA-I (30 μg/ml) was performed for 3 h with cells 48 h after transfection as described in Materials and Methods. Means ± SD of quadruplicate determinations are shown. An asterisk (*) indicates *p* < 0.001. (D) Immunoblotting of RAW 264.7 cells transfected with empty vector (mock), WT Nef derived from HIV-1 strain SF2 (Nef.wt), or Nef.G2A mutant, and stained with anti-Nef antibodies.

### Nef Is Critical for Cholesterol Efflux Impairment

Previous studies demonstrated that the HIV-1 protein Nef can directly bind cholesterol and suggested that in CD4^+^ T cells, Nef may be involved in transporting cholesterol to the sites of HIV assembly at the plasma membrane [5]. To test the role of Nef in the observed impairment of cholesterol efflux from macrophages, we infected macrophages with HIV-1 SF2 constructs carrying a mutated or a functional *Nef* gene. To ensure similar levels of infection, the constructs were pseudotyped by the glycoprotein of vesicular stomatitis virus (VSV-G), which targets HIV-1 entry to an endocytic pathway, thus eliminating the requirement for Nef in the early steps of infection [31]. Under these one-cycle replication conditions, both viruses infected about 40% of cells and produced similar levels of p24 (Figure 1B). Cholesterol efflux to apoA-I was substantially reduced in the culture infected with the Nef-positive virus (wild type [WT]), whereas in the culture infected with Nef-deficient virus (ΔNef), it was similar to the level observed in uninfected cells (Figure 1B). This result indicates that Nef is necessary for HIV-mediated impairment of cholesterol efflux.

To determine whether Nef is sufficient for the observed effect, we transiently transfected murine macrophages RAW 264.7 with constructs expressing SF2-derived Nef and stimulated ABCA1 expression in these cells by liver X receptor (LXR) agonist TO-901317. Cholesterol efflux to apoA-I from *Nef*-transfected RAW 264.7 macrophages was significantly reduced (by more than 50%) compared to cells transfected with an empty vector (mock transfection) (Figure 1C). This result indicates that expression of Nef is sufficient to impair cholesterol efflux from macrophages. Interestingly, Nef mutant Nef.G2A, defective in myristoylation and membrane localization [5], was not effective in impairing cholesterol efflux (Figure 1C) despite levels of expression being similar to that of WT Nef (Figure 1D). Cholesterol efflux impairment in Nef-transfected RAW cells was less than in HIV-infected macrophages, likely due to differences between these cell types.

### Nef Specifically Targets ABCA1-Dependent Cholesterol Efflux

Specific efflux of cholesterol from cells is mediated by the members of the family of ATP-binding cassette (ABC) transporters. The ABCA1 transporter is responsible for lipidation of lipid-poor apoA-I with cellular lipids [32], whereas ABCG1 controls efflux to mature HDL [33]. Our finding that Nef inhibits cholesterol efflux to apoA-I (Figure 1) suggests that ABCA1 may be the specific target of Nef. Consistent with this notion, cholesterol efflux to HDL (controlled by ABCG1) from HIV-infected macrophages was not significantly impaired (Figure 2A). Furthermore, phospholipid efflux, which is dependent on ABCA1 [34], from *Nef*-transfected RAW 264.7 macrophages was reduced (Figure 2B), similar to the effect of Nef on cholesterol efflux (Figure 1C). In addition, Nef did not affect cholesterol efflux from RAW 264.7 cells in which ABCA1 expression was not stimulated (Figure 2C) and consequently was very low.

**Figure 2:**
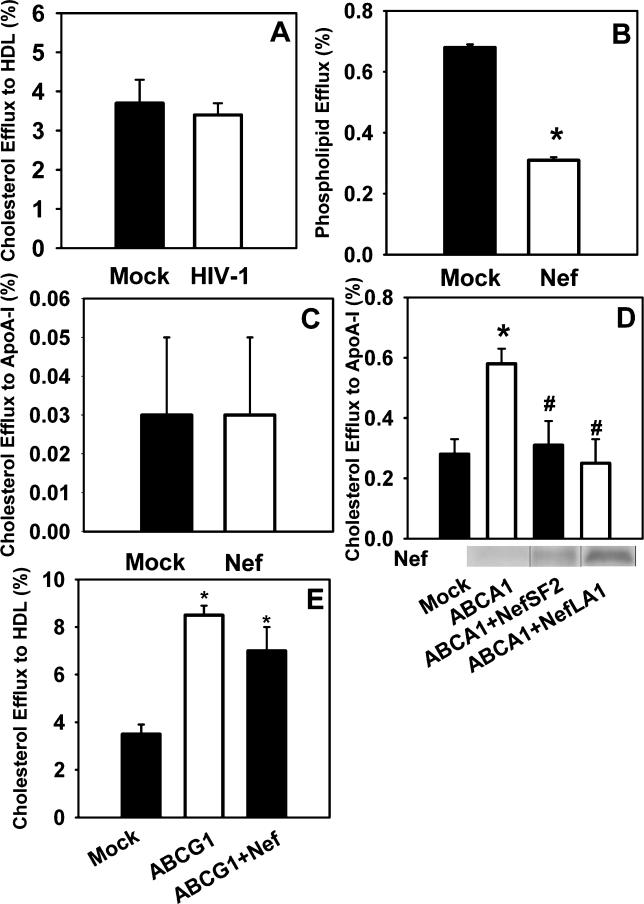
Nef Targets ABCA1-Dependent Cholesterol Efflux (A) Cholesterol efflux to HDL (30 μg/ml) was measured from HIV-1 ADA-infected and mock-infected macrophages used also to measure efflux to apoA-I in Figure 1A. (B) Impairment of phospholipid efflux in Nef-transfected RAW 264.7 cells. RAW 264.7 cells were transfected with plasmid expressing HIV-1 SF2-derived Nef or empty vector (mock-transfection). Phospholipid efflux to apoA-I (30 μg/ml) was measured as described in Methods. Means ± SD of quadruplicate determinations are shown. An asterisk (*) indicates *p* < 0.001. (C) Nef does not decrease cholesterol efflux in RAW 264.7 cells not treated with LXR agonist. Experiment was performed using HIV-1 SF2-derived Nef as described in Figure 1C, except that LXR agonist was not added. (D) Cholesterol efflux to apoA-I from HeLa cells. HeLa cells were either mock-transfected (mock) or co-transfected with ABCA1 and empty vector (ABCA1), or vector expressing Nef derived from HIV-1 SF2 (ABCA1 + NefSF2) or LAI strains (ABCA1 + NefLAI); cholesterol efflux to apoA-I was analyzed. An asterisk (*) indicates *p* < 0.001 (versus cells without ABCA1); a number sign (#) indicates *p* < 0.001 (versus cells without Nef). Expression of Nef determined by Western blot is shown beneath the bars. (E) Cholesterol efflux to HDL from HeLa cells. Experiment was performed as in (D), except that ABCG1 was used instead of ABCA1, and HDL (30 μg/ml) instead of apoA-I was used as cholesterol acceptor. An asterisk (*) indicates *p* < 0.01 (versus cells without ABCG1).

The notion that ABCA1-mediated efflux is the target of Nef was further supported by experiments in HeLa cells, which do not express ABC transporters and have very low background cholesterol efflux to apoA-I [35]. Consistent with a previous report [36], transfection of HeLa cells with *ABCA1* significantly enhanced cholesterol efflux to apoA-I, whereas co-transfection with *Nef* derived from SF2 or LAI strains of HIV-1 brought the efflux back to the level observed in mock-transfected cells (Figure 2D). Importantly, NefLAI, which was expressed to higher levels than NefSF2 (Figure 2D), was more effective also in cholesterol efflux impairment. A similar experiment testing the effect of Nef on ABCG1-directed cholesterol efflux showed stimulation of cholesterol efflux to HDL after transfection of HeLa cells with ABCG1, but did not reveal significant inhibition by Nef (Figure 2E). Therefore, we conclude that Nef specifically targets ABCA1-dependent cholesterol efflux.

### Nef Down-Regulates ABCA1

Since ABCA1 appears to be the target of Nef, we tested ABCA1 abundance (by Western blotting) and transcription (by real-time RT-PCR) in HIV-infected human macrophages and *Nef*-transfected RAW 264.7 cells. Analysis of ABCA1 in macrophages at the peak of HIV-1 ADA replication showed a substantial decrease of ABCA1 abundance (Figure 3A). Importantly, abundance of two other proteins involved in cholesterol efflux to HDL, ABCG1 and scavenger receptor B1 (SR-B1), was not affected by HIV infection (Figure 3A), consistent with specific targeting of the ABCA1-dependent pathway by the virus. A similar phenomenon was observed in Nef-transfected RAW 264.7 macrophages, although the effect was less pronounced (approximately 50% reduction in ABCA1 abundance when assessed by densitometry of the Western blot) (Figure 3B). The Nef.G2A mutant, which was inactive in cholesterol efflux impairment (Figure 1C), was also inactive in depleting ABCA1. RT-PCR analysis revealed a significant increase of ABCA1 mRNA in HIV-infected (Figure 3C) or Nef-transfected (Figure 3D) cells, which likely reflects a compensatory response for the loss of ABCA1 [37]. This observation rules out a suppressive effect of Nef on ABCA1 transcription and suggests a post-transcriptional down-regulation of ABCA1 by Nef. Therefore, down-regulation of ABCA1 is one of the mechanisms responsible for impairment of cholesterol efflux by HIV-1.

**Figure 3:**
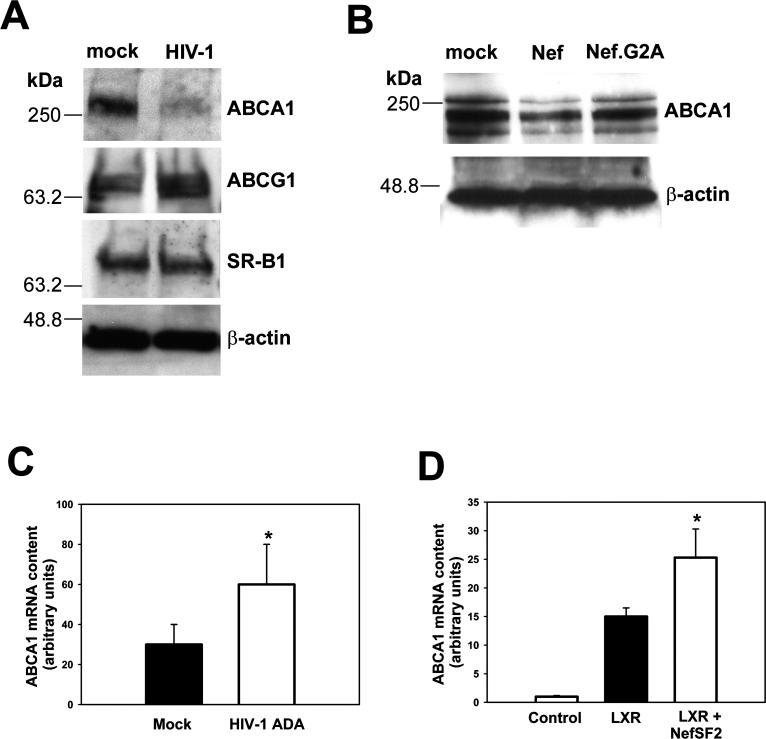
Nef Induces Down-Modulation of ABCA1 (A) Human monocyte-derived macrophages were infected with HIV-1 ADA or mock-infected and cultured for 14 d (RT in culture supernatant was 4,000 cpm/μl). ABCA1, ABCG1, SR-B1, and β-actin (loading control) were analyzed by Western blotting. (B) RAW 264.7 cells were transfected with vector expressing HIV-1 SF2-derived Nef (either WT or carrying a G2A mutation) or empty vector (mock). Twenty-four hours after transfection, cells were stimulated with TO-901317 (1 μM) and 24 h later, were analyzed by Western blotting for ABCA1 and β-actin (loading control). (C) ABCA1 RNA from HIV-infected macrophages used for Western blotting in (A) was analyzed by real-time RT-PCR. Results were adjusted according to β-actin signal and are presented in arbitrary units; an asterisk (*) indicates *p* < 0.01 (versus mock). (D) RNA was extracted from non-activated RAW 264.7 cells (control), mock-transfected RAW cells activated with LXR agonist TO-901317 (LXR), or cells transfected with SF2-derived Nef and activated with TO-901317 (LXR+NefSF2), and analyzed by real-time RT-PCR. Results were adjusted according to 28S RNA signal and are presented in arbitrary units; an asterisk (*) indicates *p* < 0.01 (versus LXR agonist-treated, mock-transfected cells).

### Nef Alters Intracellular Distribution of ABCA1

Although down-regulation of ABCA1 alone would account for a substantial part of the inhibition of cholesterol efflux, we further found that intracellular distribution of ABCA1 was also affected by HIV infection. Recent reports established that ABCA1 resides both on the plasma membrane and in endocytic vesicles [36], and demonstrated the role of endosomal ABCA1 and trafficking of ABCA1 between endosomes and plasma membrane in the apoA-I–mediated efflux of cellular lipids from the endosomal compartment [38,39]. Figure 4A and 4B show p24 staining, and Figure 4C and 4D show ABCA1 distribution in human macrophages infected with ΔNef and Nef-expressing HIV-1, respectively. Consistent with the findings of Neufeld and colleagues [36], ABCA1 was distributed evenly between the cytoplasm and the plasma membrane in human macrophages either uninfected (unpublished data) or infected with ΔNef HIV-1 (Figure 4C), as well as in mock-transfected murine RAW 264.7 cells (Figure 4E). It appears that in macrophages infected with Nef-expressing HIV-1, ABCA1 was re-localized to the cell periphery (p24^+^ cells in Figure 4D). This re-localization of ABCA1 to the plasma membrane was even more pronounced in Nef-transfected murine macrophages RAW 264.7 (compare Figure 4E and 4F). No re-localization of ABCA1 was observed in macrophages transfected with Nef.G2A (Figure 4G). Therefore, Nef expression induces re-localization of ABCA1, which requires myristoylation of Nef.

**Figure 4:**
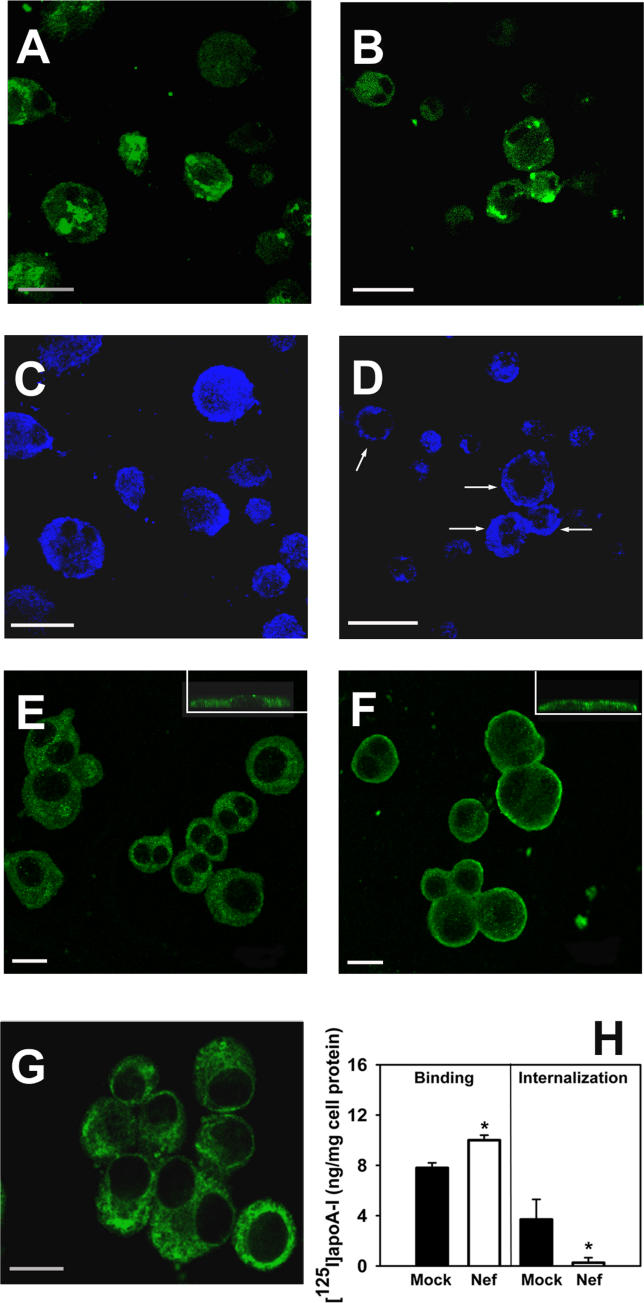
The Effect of Nef on ABCA1 Localization (A–D) On day 5 after infection with VSV-G-pseudotyped Nef-expressing (B and D) or ΔNef (A and C) HIV-1 SF2, cells were co-stained with anti-p24 mouse monoclonal and anti-ABCA1 rabbit polyclonal antibodies, followed by FITC-conjugated anti-mouse (A and B) and Cy5-conjugated anti-rabbit IgG (C and D). Arrows point to cells with re-localized ABCA1. The scale bars represent 20 μm. (E–G) Distribution of ABCA1 revealed by staining with monoclonal anti-ABCA1 antibody and FITC-conjugated anti-mouse IgG in RAW 264.7 cells transfected with empty vector (E), WT Nef derived from SF2 HIV-1 (*Nef.wt,* panel [F]), or SF2 Nef carrying a G2A mutation (*Nef.G2A*, [G]). Insets in (E and F) show cross-section of the image reconstituted from serial sectioning. Scale bars represent 20 μm. (H) [^125^-I]apoA-I binding (left panel) and internalization (right panel) in RAW 264.7 macrophages transfected with HIV-1 SF2-derived Nef. An asterisk (*) indicates *p* < 0.01.

Previous studies demonstrated that apoA-I specifically binds to ABCA1 at the cell surface [40–42]. It was also suggested that trafficking of apoA-I to intracellular cholesterol pools correlates with trafficking of ABCA1 [39,43]. Consistent with re-localization of ABCA1 to the plasma membrane, the specific binding of [^125^I]apoA-I to Nef-transfected RAW 264.7 macrophages was increased (Figure 4H, left panel). However, internalization of [^125^I]apoA-I was almost completely blocked, supporting the model whereby Nef impairs intracellular trafficking of ABCA1 (Figure 4H, right panel). Degradation of [^125^I]apoA-I was negligible and was not affected by Nef (unpublished data).

Therefore, Nef-dependent changes in intracellular distribution of ABCA1 may be another mechanism responsible for impairment of cholesterol efflux.

### Nef Interacts with ABCA1

Nef has been shown to modulate expression of several trans-membrane proteins. In some cases (e.g., with CD4 or major histocompatibility complex [MHC] I) Nef down-regulates the protein, and in some (e.g., with invariant chain of MHC class II or with dendritic cell-specific ICAM-3-grabbing nonintegrin [DC-SIGN]), it up-regulates the protein's surface expression ([44] and references therein). Some of these effects, including down-regulation of CD4 [45] and MHC I [46], have been shown to depend on an interaction between Nef and the target protein. We therefore tested whether Nef interacts with ABCA1. HeLa cells were co-transfected with Nef or Nef.G2A and FLAG-tagged ABCA1, ABCA1 was immunoprecipitated using anti-FLAG antibody, and immunoprecipitates were analyzed for co-precipitation of Nef. This analysis revealed that Nef co-precipitated with ABCA1, whereas Nef.G2A did not (Figure 5A, upper panel) despite equally high expression of the two forms of Nef (Figure 5A lower panel). We conclude that Nef can interact with ABCA1, and this interaction requires myristoylation of Nef and correlates with the ability of Nef to impair cholesterol efflux. The Nef-specific signal observed in this experiment required high-level expression of participating proteins, likely due to the transitory nature of Nef interaction with ABCA1.

**Figure 5:**
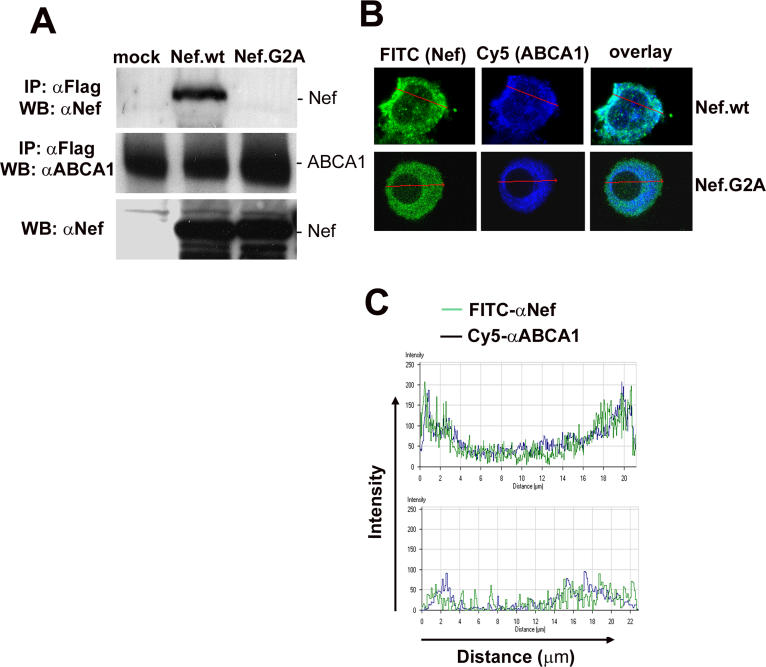
Nef Interacts with ABCA1 (A) ABCA1 was immunoprecipitated using anti-FLAG M2 affinity gel from HeLa cells co-transfected with ABCA1-FLAG and an empty vector (mock), WT SF2 Nef (Nef.wt), or myristoylation-defective mutant Nef.G2A. Immunoprecipitates were analyzed by Western blotting for Nef (upper panel) or ABCA1 (middle panel) using specific antibodies. Bottom panel shows Nef expression in lysates of cells used for immunoprecipitation. (B) Experiment was performed as in Figure 4E, except that cells were incubated with monoclonal anti-ABCA1 and polyclonal anti-Nef antibody, followed by FITC-conjugated anti-rabbit and Cy5-conjugated anti-mouse antibodies. Since all transfected cells show re-localization of ABCA1 (Figure 4E), a typical single cell is shown here. Images were analyzed using software on the Zeiss LSM 510 microscope. The scale bar represents 20 μm. (C) Fluorescence profile of the image in Figure 5B was analyzed using the LSM 510 software. The top panel shows the distributions of the ABCA1 and HIV-1 Nef proteins in blue and green, respectively. The same analysis in the lower panel was performed for ABCA1 (blue) and Nef.G2A (green). The co-localization of WT Nef and ABCA1 at the plasma membrane is indicated by overlapping green and blue peaks at either end of the graph in the top panel.

Interaction between ABCA1 and Nef at the plasma membrane was supported by confocal microscopy, which demonstrated co-localization of Nef and ABCA1 in RAW 264.7 cells transfected with Nef.wt-expressing vector (Figure 5B). No co-localization was observed between ABCA1 and Nef.G2A (Figure 5B). This visual analysis was reinforced by an analytical quantification presented in Figure 5C. Indeed, both ABCA1 and the WT Nef proteins are found at the cell periphery, and their co-localization is indicated by overlapping green and blue peaks at either end of the graph. Moreover, both colors peak and valley in tandem, suggesting a correlation in subcellular localization of ABCA1 and WT Nef. No such correlation is observed in ABCA1 and Nef.G2A distribution. Taken together, these results suggest that interaction between ABCA1 and Nef occurs at the cell plasma membrane.

Therefore, both re-localization and down-modulation of ABCA1 depend on its interaction with Nef, which in turn requires myristoylation and membrane localization of Nef.

### HIV-Infected Macrophages Transform into Foam Cells

To determine whether impairment of cholesterol efflux by HIV-1 infection leads to cholesterol accumulation and foam cell formation, we loaded macrophages (uninfected or infected with Nef-expressing or ΔNef HIV-1) with lipids by incubating with acetylated LDL (AcLDL) in the presence of apoA-I and stained cellular lipids with Oil Red O (Figure 6). This experiment revealed formation of typical lipid-rich cells in cultures infected with Nef-expressing HIV-1, whereas uninfected cells or macrophages infected with ΔNef virus accumulated substantially less cholesterol (compare Figure 6A, 6B, and 6C). Analysis by transmission electron microscopy revealed more lipid vacuoles (arrows in Figure 6E) in macrophages infected with Nef-expressing HIV-1 than in uninfected cells or cells infected with ΔNef HIV-1 (compare Figure 6D, 6E, and 6F). Cholesterol loading of RAW 264.7 macrophages transfected with Nef also led to accumulation of significantly larger amounts of lipids when compared to cells transfected with an empty vector (compare Figure 6H and 6I). In addition, Nef-transfected RAW 264.7 macrophages demonstrated accelerated cholesteryl ester synthesis, especially when cells were loaded with AcLDL (Figure 6G). Enhanced synthesis of cholesteryl esters is a sensitive indicator of accumulation of cholesterol inside the cells and a key element of foam cell formation.

**Figure 6:**
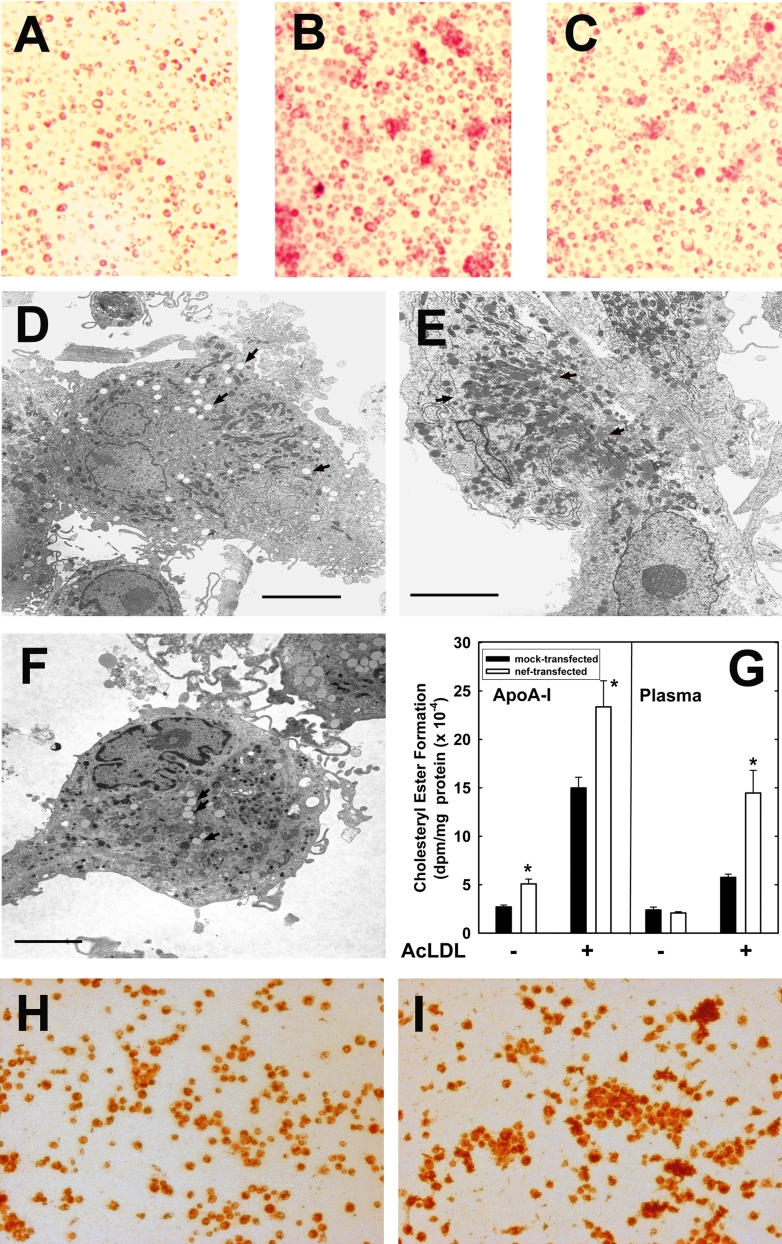
Accumulation of Lipids in Cells Infected with HIV-1 or Transfected with Nef (A–C) Oil Red O staining of HIV-infected macrophages. Uninfected (A) macrophages or cells infected with VSV-G–pseudotyped Nef-positive (B) or ΔNef (C) HIV-1 SF2 variants were loaded with cholesterol on day 3 after infection by incubating with AcLDL in the presence of apoA-I, and lipids were stained with Oil Red O 24 h later. p24 concentration in the culture supernatant on day 3 after infection was 4.7 ng/ml for cells inoculated with Nef-positive virus and 9.8 ng/ml for the culture inoculated with ΔNef HIV-1. (D–F) Electron microscopy of cholesterol-loaded uninfected macrophages (D) and cells infected with Nef-positive (E) and ΔNef (F) HIV-1 AD8 performed 14 d after infection. Uninfected cells have small numbers of electron-lucent lipid vacuoles (arrows). The cytoplasm of cells infected with Nef-positive virus is filled with electron-dense lipid vacuoles (arrows). Cells infected with ΔNef virus have small numbers of electron-lucent lipid vacuoles (arrows), similar in number to those in uninfected cells. The scale bars represent 5 μm. (G) The effect of Nef on cholesteryl ester synthesis. The rate of cholesteryl ester synthesis in RAW 264.7 cells transfected with an empty vector (mock-transfected) or Nef-expressing construct and incubated with or without AcLDL in the presence of apoA-I or 5% human plasma is presented as mean ± SD of quadruplicate determinations. An asterisk (*) indicates *p* < 0.02. (H) and (I) RAW 264.7 cells were transfected with empty vector (H) or Nef-expressing construct (I), stimulated with LXR agonist, incubated with AcLDL and lipid-free apoA-I, fixed with formaldehyde, and stained with Oil Red O.

Measurements of cholesterol mass confirmed substantially higher cholesteryl ester content in Nef-transfected RAW 264.7 macrophages compared to mock-transfected cells (Figure 7A); there was also more free cholesterol in the transfected macrophages (Figure 7B). Synthesis of triglycerides was not affected (Figure 7C), indicating that increased cholesteryl ester synthesis and content is a consequence of increased concentration of cholesterol rather than of fatty acids. The increased cholesterol content in Nef-transfected cells was not caused by differences in AcLDL uptake, as the latter was similar between Nef-transfected and mock-transfected cells (Figure 7D). To accommodate the increasing amounts of cholesteryl esters, cells would require an additional amount of phospholipids, and, indeed, the efflux of phospholipids was inhibited (Figure 2A), whereas phospholipid synthesis was accelerated in Nef-transfected cells (Figure 7E). Taken together, these results indicate that HIV-1 infection, via Nef expression, impairs reverse cholesterol transport in macrophages and leads to accumulation of lipids and formation of foam cells.

**Figure 7:**
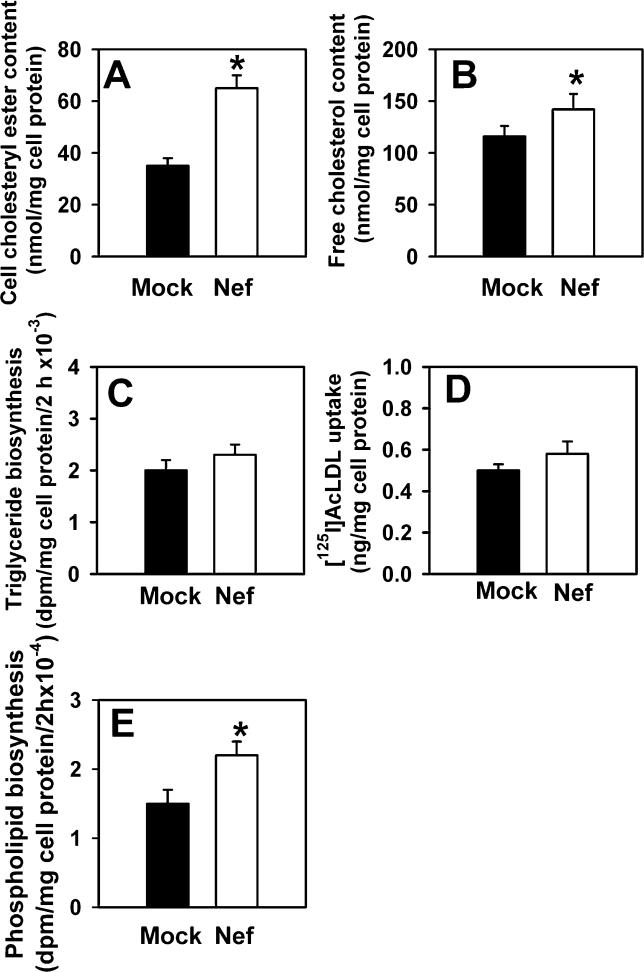
Analysis of Lipids in RAW 264.7 Macrophages Transfected with Nef (A) Cholesteryl ester content after 24 h incubation with AcLDL (50 μg/ml) determined by enzymatic assay; an asterisk (*) indicates *p* < 0.01. (B) Free cholesterol content after 24 h incubation with AcLDL (50 μg/ml) determined by enzymatic assay; an asterisk (*) indicates *p* < 0.05. (C) Triglyceride biosynthesis after 24 h incubation with AcLDL (50 μg/ml) measured as incorporation of [^14^C]oleic acid into triglycerides as described in Materials and Methods. (D) Uptake of AcLDL was calculated as a sum of ^125^I-AcLDL specifically taken up and degraded by cells. (E) Phospholipid biosynthesis measured as incorporation of [^14^C]choline into phospholipid fraction as described in Materials and Methods; an asterisk (*) indicates *p* < 0.01.

### HIV-Positive Foam Cells in Atherosclerotic Plaques of HIV-Infected Patients

Our finding that HIV-1 infection of macrophages impairs cholesterol efflux from these cells suggests that HIV-infected macrophages may potentially contribute to the development of atherosclerotic plaques, especially when combined with dyslipidemia found in PI-treated patients. Immunostaining of sections of atherosclerotic plaques obtained from highly active antiretroviral therapy (HAART)-treated HIV-infected patients demonstrated the presence of p24^+^ macrophages (Figure 8A, 8B, 8E, and 8F). In areas surrounding lipid cores, some p24^+^ cells displayed a typical foam cell appearance (Figure 8B). Analysis of parallel consecutive sections stained with anti-CD68 showed that these p24^+^ cells were located in areas composed of CD68^+^ cells (Figure 8C), indicating the macrophage nature of p24^+^ foam cells. Double immunostaining confirmed this notion by demonstrating the association of p24 staining with CD68^+^ macrophages and macrophage foam cells (Figure 8E and 8F). These findings indicate that HIV-infected, cholesterol-loaded macrophages are present in the atherosclerotic plaque and therefore may potentially be involved in pathophysiological events leading to the development of atherosclerosis.

**Figure 8:**
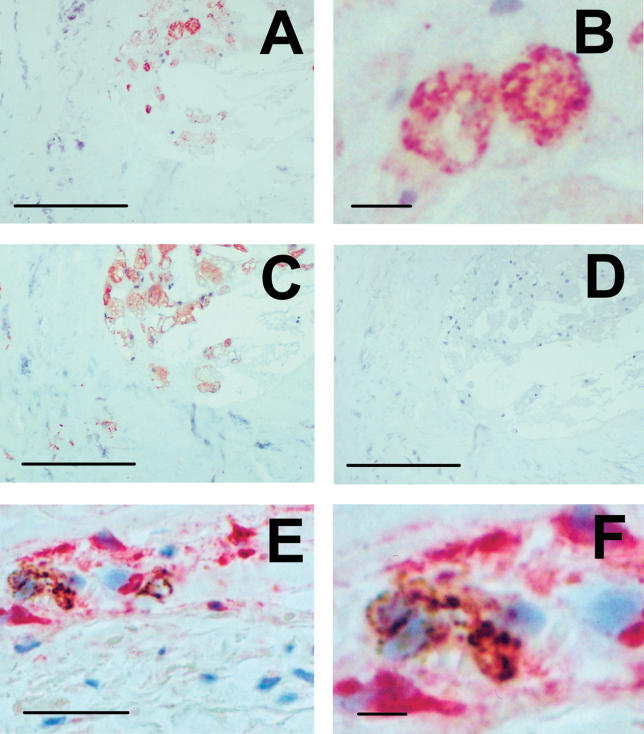
Identification of HIV-1–Positive Macrophages in Atherosclerotic Plaques of HIV-Infected Subjects. Single (A–D) and double (E and F) immunostaining of aortic wall segments. (A) p24 staining. A low-magnification image showing the presence of p24^+^ cells in an area adjacent to the plaque lipid core. The scale bar represents 100 μm. (B) Detail of (A). p24^+^ cells show a characteristic morphology of foam cells. The scale bar represents 10 μm. (C) CD68 staining. CD68^+^ cells were identified in a parallel consecutive section to that shown in (A). The scale bar represents 100 μm. (D) Negative control (staining with an irrelevant primary antibody). The scale bar represents 100 μm. (E) Double immunostaining showing the co-localization of p24 (brown) with CD68 (rose). Immunostaining included a combination of a rabbit polyclonal anti-p24 antibody in the peroxidase–anti-peroxidase system with DAB chromogen yielding a brown reaction product, and a mouse monoclonal antibody to CD68 in the alkaline phosphatase–anti-alkaline phosphatase system with Fast Red chromogen, resulting in a rose precipitate. Counterstaining was with Mayer's hematoxylin. The scale bar represents 50 μm. (F) A detail of (E). The scale bar represents 15 μm.

### Active Cholesterol Efflux Reduces Infectivity of HIV Virions

To determine whether impairment of cholesterol efflux has a role in HIV biology, we compared infectivity of HIV virions produced from monocyte-derived macrophages stimulated or not with an LXR agonist, TO-901317. We hypothesized that if impairment of cholesterol efflux is a specific mechanism to increase HIV replication, then agents counteracting this effect should have anti-HIV activity. LXR agonists up-regulate expression of ABCA1 at a transcriptional level and stimulate cholesterol efflux from various cell types, including human monocyte-derived macrophages ([47] and Figure 9A). When added to HIV-infected macrophages at day 7 after infection and kept with cells for another 7 d (to allow ABCA1 to accumulate and overcome Nef-mediated inhibition), LXR agonist prevented the impairment of cholesterol efflux by HIV-1 infection (Figure 9A); in fact, cholesterol efflux from TO-901317–treated HIV-infected macrophages was similar to efflux from uninfected cells stimulated with the LXR agonist. The lack of HIV-specific reduction of cholesterol efflux is likely due to overproduction of ABCA1, which exceeds production of Nef. Virions were collected from TO-901317–treated and untreated cells, adjusted according to p24 content, and analyzed for infectivity using indicator P4-CCR5 cells. This analysis revealed a substantial reduction (by about 80%) of infectivity of virions produced from macrophages treated with LXR agonist (Figure 9B). Interestingly, protein composition of the virions produced from LXR agonist–treated and untreated cells was very similar (unpublished data), whereas virion-associated cholesterol was significantly diminished in virions produced from TO-901317–treated macrophages (Figure 9C). These results suggest that stimulation of cellular cholesterol efflux may be an effective approach to suppressing HIV replication.

**Figure 9:**
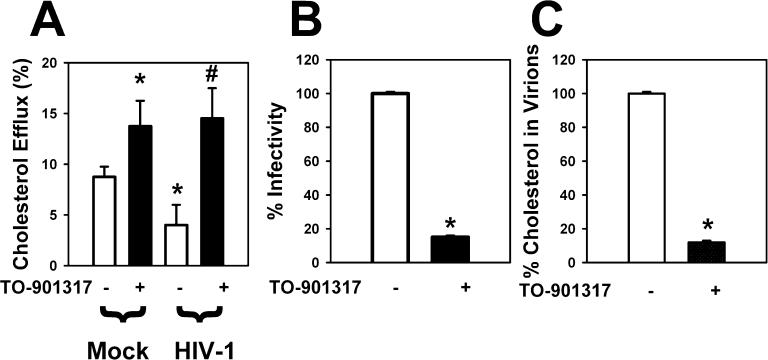
Cholesterol Efflux and Infectivity of HIV Virions Human monocyte-derived macrophages were infected with HIV-1 ADA or mock-infected, and 7 d after infection were treated or not treated with LXR agonist, TO-901317 (500 nM), for seven more days. (A) Cholesterol efflux to apoA-I was measured on day 21 after infection. An asterisk (*) indicates *p* < 0.01 (versus uninfected cells not treated with TO-901317); a number sign (#) indicates *p* < 0.01 (versus HIV-infected cells not treated with TO-901317). (B) Virions were collected from culture supernatants of LXR agonist-treated and untreated (control) cells on day 10 and day 14 (pooled together), adjusted according to p24 content, and analyzed for infectivity on indicator P4-CCR5 cells. Experiment was performed in triplicate, and results (mean ± SD) are presented as percent infectivity of virions produced by control cells; an asterisk (*) indicates *p* < 0.001. (C) Incorporation of [^3^H]cholesterol into virions produced by LXR agonist-treated and untreated (control) cells was measured in triplicate, and results (mean ± SD) are presented relative to cholesterol in the virions produced by control cells; an asterisk (*) indicates *p* < 0.001.

## Discussion

Results presented in this report demonstrate that HIV-1, via the accessory protein Nef, impairs cholesterol efflux from macrophages. This finding can be interpreted as a virus-mediated switch of cholesterol trafficking from physiological efflux to virus-controlled transport, thus reducing the ability of a cell to export excessive cholesterol. Given that availability of cholesterol is critical for HIV assembly and infectivity [48], it is physiologically sensible for the virus to take over control of intracellular cholesterol metabolism.

A previous report demonstrated that Nef binds cholesterol and may deliver it to nascent virions [5]. Our study suggests that Nef-mediated impairment of cholesterol efflux is another mechanism ensuring efficient delivery of cholesterol to HIV. Importantly, this mechanism may be a necessary component of the above-mentioned Nef-mediated transport of cholesterol to virions. Indeed, prevention of cholesterol efflux impairment by LXR agonist reduces virion-associated cholesterol without interfering with Nef incorporation into the virions (unpublished data). Reduction of virion-associated cholesterol correlates with lower virion infectivity (Figure 9B and 9C).

Our results demonstrate that Nef specifically targets ABCA1. Indeed, Nef did not suppress cholesterol efflux in cells lacking ABCA1 (HeLa cells or non-activated RAW 264.7 cells), but did so in ABCA1-expressing cells, such as RAW 264.7 cells stimulated with an LXR agonist, HeLa cells transfected with ABCA1, and differentiated human macrophages. Furthermore, Nef did not suppress cholesterol efflux from ABCG1-transfected HeLa cells (Figure 2E). These findings and the fact that Nef can interact with ABCA1 (Figure 5A) suggest that there is an interplay between Nef and ABCA1 in an HIV-infected cell. The end result of this interplay would depend on relative levels of expression of Nef and ABCA1. Consistent with this suggestion, overexpression of Nef from the cytomegalovirus (CMV) promoter inhibits ABCA1-mediated cholesterol efflux stimulated with the LXR agonist (Figure 1C), whereas levels of Nef expressed from the HIV LTR are insufficient to suppress LXR agonist-stimulated cholesterol efflux in HIV-infected macrophages (Figure 9A). As a result, HIV infectivity is reduced in LXR agonist-stimulated cells (Figure 9B). Therefore, drugs stimulating cholesterol efflux may provide a dual benefit to HIV-infected patients by limiting HIV replication and reducing the risk of atherosclerosis.

Our studies show that cholesterol efflux impairment is a conserved feature of HIV-1 Nef. Indeed, we show this phenomenon using three R5 (ADA, Yu-2, and 92US660) and two X4 (SF2 and LAI) HIV-1 isolates. We demonstrate that HIV-1 Nef impairs cholesterol efflux by at least two mechanisms: it reduces ABCA1 abundance, and it causes intracellular re-distribution of ABCA1. These two mechanisms may be related, as they both depend on interaction between Nef and ABCA1 (Figures 3–5). For example, a block to intracellular trafficking of ABCA1 may re-target this protein to a degradation pathway. Alternatively, ABCA1 re-distribution and down-regulation may be two independent effects of Nef, both contributing to impairment of cholesterol efflux. Indeed, several recent reports demonstrated a role of ABCA1 trafficking between late endosomes and the cell surface in cholesterol efflux from endosomal compartment [36,38,39,49]. The effects of Nef on ABCA1 distribution and apoA-I binding and internalization are similar to the effects of cyclosporin A [43] or deletion of the PEST sequence in ABCA1 [39], both of which inhibit efflux of cholesterol from late endosomes. Therefore, both down-regulation of ABCA1 and its intracellular re-distribution can independently contribute to cholesterol efflux impairment observed in HIV-infected cells. Interestingly, Nef alone had less effect on ABCA1 abundance than HIV-1 infection (compare Figure 3A and 3B), however, it had a more profound effect on the sequestration of ABCA1 at the plasma membrane (compare Figure 4D and 4F). Although this result may be due to differences between the cell types in which these analyses were performed (primary human macrophages for HIV infection and murine macrophage cell line RAW 264.7 for *Nef* transfection), it is also possible that the primary effect of Nef is to sequester ABCA1 at the plasma membrane, and some other HIV protein may cooperate with Nef to stimulate down-regulation of sequestered ABCA1.

The exact molecular mechanisms responsible for the effect of Nef on intracellular trafficking and abundance of ABCA1 are yet to be fully investigated. Nef is known to regulate expression of several transmembrane proteins, including CD4 [45], MHC I [50], MHC II [50], CD28 [51], and DC-SIGN [52]. In most cases, Nef mediates internalization and degradation of the receptor [53], but in some cases (e.g., with DC-SIGN or invariant chain of MHC II), it up-regulates the cell surface expression of the protein. These effects are consistent with our findings showing ABCA1 re-localization and down-regulation, which may involve mechanisms similar to those described for Nef interactions with other proteins. It is worth noting that few of the above-mentioned studies showed co-immunoprecipitation of Nef with a target protein from HIV-infected cells, consistent with our inability to pull down Nef and ABCA1 from HIV-infected macrophages. This may be due to a transitory nature of Nef–ABCA1 interaction and low-level expression of these proteins. Analysis of this interaction in cells overexpressing both proteins demonstrated a critical role of Nef myristoylation (Figure 5A). This fatty acid may either be directly involved in binding of Nef to ABCA1, similar to the role that farnesylation of the yeast pheromone a-factor plays in its interaction with the yeast ABC transporter Ste6 [54], or it may regulate Nef–ABCA1 interaction indirectly by targeting Nef to the plasma membrane. Further analysis of the mechanisms by which Nef affects ABCA1 function would require understanding of the molecular events that regulate intracellular trafficking and degradation of ABCA1 in uninfected cells, which is incompletely characterized and is a subject of the ongoing studies.

The results of this study have potential implications for understanding pathogenesis of CAD in HIV-infected patients. These patients have a mildly elevated risk of CAD [55], which is sharply raised by treatment with certain PIs [7,9,14,19]. Increased risk of CAD after treatment with PIs led to the assumption that PIs and/or dyslipidemia are the primary source of development of atherosclerosis in HIV patients. Results presented in this report suggest that HIV-induced impairment of cholesterol efflux from macrophages may be another important contributor to the pathogenesis of CAD. Indeed, inactivation of ABCA1 in macrophages of hyperlipidemic mice significantly increased development of atherosclerosis [27], and genetic mutation inactivating ABCA1 in humans leads to Tangier disease, one of the characteristic features of which is an increased risk of CAD [28]. Impairment of reverse cholesterol transport mediated by down-regulation of ABCA1 has been described for bacterial infections and has been linked to pathogenesis of atherosclerosis (reviewed in [56]). In the case of HIV infection, this mechanism would have only a mild atherogenic effect or not at all on the background of hypocholesterolemia characteristic for untreated HIV-1 infection [55,57]. Treatment of HIV-infected patients with HAART causes a sharp rise of triglyceride-rich VLDL, resulting in enhanced lipid uptake and foam cell formation [58], and small dense LDL [59,60], which is particularly susceptible to oxidation [61], is more able to infiltrate the subendothelial space, and is a risk factor for CAD [62]. A combination of these effects of HAART and impairment of cholesterol efflux by HIV (which prevents compensatory removal of excessive cholesterol) would result in a greatly enhanced accumulation of cholesterol in HIV-infected macrophages and would potentially further increase the risk of development of atherosclerosis. It should be noted that HIV-infected macrophages, unlike T cells, survive for extended periods of time and are considered long-term reservoirs of HIV-1 [63]. As a result, infected macrophages persist, at least for some time, in HAART-treated patients, when conditions favor development of atherosclerotic plaques. We can speculate that these macrophages may contribute to initiation of atherosclerotic plaque formation, which then proceeds even in the absence of newly infected cells. This mechanism is consistent with the presence of HIV-infected macrophages in atherosclerotic plaques of HAART-treated patients observed in our study (Figure 8). However, further in vivo and clinical studies are required to evaluate the contribution of the impairment of reverse cholesterol transport to the risk of atherosclerosis in HIV patients.

Findings presented in this report provide an example of how viruses may interfere with cellular cholesterol metabolism and may potentially affect the risk of atherosclerosis. This example may be not unique to HIV. Other viruses (such as Herpes virus or CMV) were found in atherosclerotic plaques and were epidemiologically associated with elevated risk of development of atherosclerosis [64–66]. Future studies will determine whether these viruses cause disturbances in cholesterol metabolism similar to those found in this report. In support of this possibility, several reports demonstrated that bacterial and viral pathogens may modulate macrophage cholesterol efflux by down-regulating ABC transporters via LXR-dependent [24] and LXR-independent [25] pathways. The first pathway is engaged after activation of Toll-like receptors by invading viruses or bacteria. The second pathway involves the negative effect of bacterial endotoxin on ABCA1 mRNA levels in macrophages [25]. Both pathways promote conversion of macrophages into foam cells, which may acquire resistance to pathogen, but retain their atherogenic properties [12]. Therefore, the effect on reverse cholesterol transport may be a common feature of viral and bacterial infection of macrophages, although mechanisms involved are likely unique for each infection.

## Materials and Methods

### Human monocyte-derived macrophage cultures.

Monocyte-derived macrophages were prepared from peripheral blood mononuclear cells of normal donors using adherence to plastic, and differentiated in the presence of macrophage colony-stimulating factor essentially as previously described [67]. No stimulation with LXR agonist was performed unless indicated.

### Antibodies.

The following antibodies were used for Western blotting: rabbit polyclonal anti-ABCA1 (Novus Biologicals, Littleton, Colorado, United States), rabbit polyclonal and mouse monoclonal anti-Nef (AIDS Research and Reference Reagent Program), rabbit polyclonal anti-ABCG1 (Novus Biologicals), rabbit polyclonal anti-SR-B1 (Novus Biologicals), and mouse monoclonal anti-β-actin (Sigma, St. Louis, Missouri, United States).

### Viruses and infections.

Macrophage-tropic HIV-1 strains Yu-2 and 92US660 were obtained from the National Institutes of Health (NIH) AIDS Research and Reference Reagent Program. VSV-G–pseudotyped HIV-1 was prepared by co-transfecting HEK 293T cells with Env-deficient HIV-1 infectious clones [5] and VSV-G-expressing plasmid pHEF-VSVG [68]. All infections were performed using 3.5 × 10^6^ cpm of RT activity per 10^6^ cells.

### FACS analysis of HIV-infected macrophages.

To determine the percentage of HIV-infected cells, HIV-infected macrophages were detached from the plate, fixed in 4% formalin/PBS, permeabilized using Becton-Dickinson permeabilization/washing solution (15 min at 4 °C), and incubated with PE-conjugated anti-p24 monoclonal antibodies (mAb) or isotype control IgG for 30 min at 4 °C. After washing, cells were analyzed on a FACScan flow cytometer (Becton-Dickinson, Palo Alto, California, United States).

### Transfection.

RAW 264.7 mouse macrophage cells were transiently transfected using the DEAE-dextran method and 5-azacytidine as described previously [69]. The efficiency of transfection was 80%–90%. HEK 293T and HeLa cells were transfected using Metafectene reagent (Biontex, Munich, Germany) following the manufacturer's protocol. The efficiency of transfection was 80%.

### Cholesterol and phospholipid efflux from RAW 264.7 cells.

RAW 264.7 cells were incubated in labeling medium containing [^3^H]cholesterol (75 kBq/ml) or [methyl-^14^C]choline (0.2 MBq/ml) for 48 h. Cells were then incubated for 18 h in serum-free medium in the presence of the LXR agonist TO-901317 (final concentration 1 μM) to stimulate ABCA1 expression and cholesterol efflux. Cells were then washed with PBS and incubated for 3 h in either serum-free medium alone (blank) or in serum-free medium supplemented with 30 μg/ml of lipid-free apoA-I or 30 μg/ml of HDL [70]. For cholesterol efflux analysis, aliquots of medium and cells were counted. Phospholipids were isolated from medium and cells by thin layer chromatography (TLC) as described previously [71]. The efflux was calculated as radioactivity in the medium/(radioactivity in the medium + radioactivity remaining in the cells) × 100%. ApoA-I–dependent efflux is efflux to apoA-I minus efflux to blank.

### Cholesterol efflux from monocyte-derived macrophages and HeLa cells.

The same procedure as described above was used except that cells were not stimulated with LXR agonist, and efflux was allowed to proceed for 12 h rather than the 3 h used for RAW 264.7 cells. Efflux to serum-free medium supplemented with 30 μg/ml of human serum albumin was used as a control.

### Phospholipid biosynthesis.

RAW 264.7 cells were incubated in serum-free medium containing [methyl-^14^C]choline (0.2 MBq/ml) for 2 h. Cells were washed, and lipids were extracted and separated by TLC [71]. Phospholipid biosynthesis was defined as incorporation of [^14^C]choline into phospholipids per mg of cell protein per 2 h.

### Cholesteryl ester and triglyceride biosynthesis and content.

RAW 264.7 cells were incubated with or without AcLDL (50 μg/ml) in the presence of 30 μg/ml of lipid-free apoA-I or 5% normolipidemic human plasma for 2 h at 37 °C. Cells were then incubated for 2 h with 37 kBq/ml [^14^C]oleic acid (presented to the cells as a BSA–sodium oleate complex). Cells were washed, lipids extracted and [^14^C]oleic acid incorporation into cholesteryl esters and triglyceride was measured after separation of the extracts by TLC [72]. Cellular free and total cholesterol content were measured using enzymatic assay (Roche, Basel, Switzerland). Free cholesterol content was calculated as a difference between total and esterified cholesterol.

### AcLDL uptake.

AcLDL was labeled with ^125^I using Iodobeads (Pierce Biotechnology, Rockford, Illinois, United States) according to the manufacturer' instructions, the final specific radioactivity was 100 cpm/ng protein. Cells were incubated with [^125^I]AcLDL at the final concentration 5 μg/ml in the presence or absence of 100-fold excess of unlabeled AcLDL for 2 h at 37 °C. The amount of degraded [^125^I]AcLDL in the medium was determined as non-iodine trichloroacetic acid–soluble radioactivity. Cells were then washed and counted, and specific AcLDL uptake was calculated as a sum of cell-associated and degraded [^125^I]AcLDL after subtraction of non-specific binding and degradation (i.e., measured in the presence of unlabeled AcLDL).

### ApoA-I binding, internalization, and degradation.

Human apoA-I was labeled with ^125^I using Iodobeads; the final specific radioactivity was 200-cpm/ng protein. Cells were incubated with [^125^I]apoA-I at the final concentration 2 μg/ml in the presence or absence of a 100-fold excess of unlabeled apoA-I for 2 h at 37 °C. The amount of degraded [^125^I]apoA-I in the medium was determined as non-iodine trichloroacetic acid–soluble radioactivity. Cells were then washed, treated with 0.05% trypsin for 5 min at 37 °C to remove surface-bound [^125^I]apoA-I, and centrifuged. Radioactivity in supernatant (binding) and pellet (internalization) was counted, and specific apoA-I binding, internalization, and degradation were calculated after subtraction of non-specific values measured in the presence of unlabeled apoA-I.

### Co-immunoprecipitation of Nef and ABCA1.

HeLa cells were co-transfected with ABCA1-FLAG and Nef. At 48-h post-transfection, cells were homogenized in PBS, and batch immunoprecipitations using M2 affinity gel (Sigma) were carried out according to the manufacturer's protocol. Immunoprecipitate was analyzed by Western blotting using polyclonal anti-ABCA1 (Novus Biologicals) and anti-Nef (AIDS Research and Reference Reagent Program) antibodies.

### Oil Red O Staining.

Human monocyte-derived macrophages and RAW 264.7 cells (stimulated with LXR agonist) were incubated with AcLDL (50 μg/ml) in RPMI 1640 supplemented with 1% Nutridoma (Roche) and 30 μg/ml of lipid-free apoA-I for 18 h. After washing with PBS, cells were fixed in 3.7% formaldehyde for 2 min, washed with water, and incubated at room temperature for 1 h with Oil Red O working solution (Fisher Biotech, West Perth, Australia). The Oil Red O solution was removed by aspiration, the cells were washed with water, and 0.02% sodium azide was added to the cells. The cells were observed using a reflecting light microscope (90× magnification) fitted with a camera.

### Fluorescent Microscopy.

For visualization of ABCA1 localization, approximately 10^6^ monocyte-derived macrophages were plated into each well of a 24-well plate containing polylysine-coated coverslips (Becton Dickinson Labware, Bedford, Massachusetts, United States) and infected with VSV-G pseudotyped WT or Nef-deleted HIV-1 viruses, or left uninfected. At 5-d post-infection, coverslips with adherent macrophages were removed from the wells and washed with PBS, and cells were fixed with 4% formaldehyde (in PBS) for 20 min at room temperature. After fixing, cells were washed three times with PBS and incubated for 1 h at room temperature in 4% goat serum (in PBS). After washing in PBS, rabbit polyclonal antibody to ABCA1 (Novus Biologicals) and mouse monoclonal antibody to p24 [73] were added in 4% goat serum, 0.1% BSA, and 0.1% Saponin. Anti-ABCA1 antibody was added at a 1:500 dilution and anti-p24 antibody, at 1:100. Following a 2-h incubation at room temperature, the coverslips were washed with PBS and incubated with Cy-5–conjugated anti-rabbit and FITC-conjugated anti-mouse secondary antibodies (Jackson ImmunoResearch, West Grove, Pennsylvania, United States) for 1 h at room temperature. Coverslips were mounted onto slides using Prolong (Molecular Probes, Eugene, Oregon, United States) and allowed to dry overnight. Visualization of stained cells was accomplished using a Bio-Rad MRC 1024 confocal laser scanning microscope and software (Bio-Rad, Hercules, California, United States).

RAW 264.7 cells (LXR agonist-stimulated) were grown on collagen-coated coverslips and transfected with Nef-expressing construct or an empty vector. Cells were cultured for 48 h prior to immunostaining, washed with PBS, fixed with 4% formaldehyde, and quenched with 50 mM NH_4_Cl. After permeabilization with 0.5% Triton X-100, cells were incubated with the monoclonal anti-ABCA1 antibody NDF4C2 for 1 h, washed with PBS, and incubated in the dark with a secondary goat anti-mouse FITC-labeled antibody for 1 h. For co-localization analysis, monoclonal anti-ABCA1 and polyclonal anti-Nef antibody were used, followed by Cy5-conjugated anti-mouse (staining ABCA1 in blue) and FITC-conjugated anti-rabbit (staining Nef in green) antibodies. Cells were washed again with PBS and, after mounting onto glass slides, were studied using Zeiss META confocal microscope (Zeiss, Oberkochen, Germany). Image analysis was performed using the LSM 510 software of the Zeiss microscope which analyzes the distribution of the fluorescence along cell section.

### Transmission electron microscopy.

Macrophages infected with HIV-1_ADA_ were fixed in 2.5% neutral-buffered glutaraldehyde, pelleted, gelled into agar, post-fixed in 1% OsO_4_, block-stained in uranyl acetate, dehydrated in graded ethanol and propylene oxide, and embedded in Spurr's resin. Thin sections were stained with uranylacetate and lead citrate, and examined on a LEO EM10 electron microscope (LEO Electron Microscopy, Thornwood, New Jersey, United States) at 60 kV.

### Real-Time RT-PCR.

cDNA was prepared from total cellular RNA from human monocyte-derived macrophages and analyzed by QPCR using IQ Sybr Green Supermix from Bio-Rad according to the manufacturer's recommendations with the following primers (300 nM of each primer per sample): ABCA1 sense, 5′-GAGCCTCCCCAGGAGTCG-3′; ABCA1 antisense, 5′-CAAACATGTCAGCTGTTACTGGAAG-3′; β-actin sense, 5′- GCCGTACCACTGGCATCGTG-3′; β-actin antisense, 5′-GTGGTGGTGAAGCTGTA-3′. Primers were ordered from Integration DNA Technologies (Coralville, Iowa, United States). Serial dilutions of ABCA1-pTRE [35] and β-actin cDNA (QPCR Plasmid Standard from Invitrogen, Carlsbad, California, United States) plasmids were used to calculate the copy number of ABCA1 and β-actin cDNA per sample, and results were adjusted according to β-actin cDNA levels. The abundance of ABCA1-specific RNA in RAW cells was determined as described previously [72].

### Autopsies.

Segments of the aortic wall were removed at autopsy from four HAART-treated AIDS patients (males, aged 39, 40, 44, and 47 y) at the Institute of Forensic Medicine, Sydney, Australia. The specimens were fixed in formalin and embedded in paraffin. Parallel sections were immunostained with the p24 antibody to detect HIV-1, and anti-CD68 antibody to identify macrophages. Single and double (using DAKO-DOUBLESTAIN Kit System 40; Dako, Glostrup, Denmark) immunostaining was carried out as described previously [74]. Sections of lymph nodes excised from the AIDS patients served as a positive control.

### Infectivity assay.

Virus infectivity was analyzed using P4-CCR5 indicator cells [75] that express β-galactosidase under control of HIV-1 LTR. Briefly, cells were seeded into a 96-well plate at a density of 7,500 cells per well and allowed to adhere overnight. The medium was then removed and replaced with virus in suspension (normalized according to RT activity) or with fresh medium as a control. Infection was allowed to proceed for 48 h at 37 °C, then the virus was removed, and 100 μl of lysis buffer (β-Galactosidase Enzyme Assay System; Promega, Madison, Wisconsin, United States) was added. The plate was then frozen overnight at −70 °C to ensure efficient lysis. Upon thawing, 50 μl of lysate was placed into a new 96-well plate, and 50 μl of 2× Assay Buffer was added. The plate was incubated at 37 °C for 1.5 h and optical density (OD) readings were taken at 420 nm.

### Analysis of virion-associated cholesterol.

Seven days post-infection with HIV-ADA, [^3^H]cholesterol was added to the cultures at a final concentration of 75 kBq/ml. At 24 h after addition of cholesterol, the labeled medium was removed, the cells were washed with PBS, and the new medium was added with or without 500 nM of LXR agonist, TO-901317 (Sigma). Culture supernatants were collected every 3 d over the period of 2 wk and pooled; virions were pelleted by ultracentrifugation and normalized according to p24 value, and the amount of incorporated [^3^H]cholesterol was counted on a beta-counter.

### Statistical analysis.

All experiments were reproduced two to four times, and representative experiments are shown. The Student *t* test was used to determine statistical significance of the differences.

## Supporting Information

### Accession Numbers

The Swiss-Prot (http://www.ebi.ac.uk/swissprot) accession numbers for the proteins discussed in this paper are ABCA1 (O95477), ABCG1 (P45844), LAI-derived Nef (P03406), and SF2-derived Nef (P03407).

## Abbreviations

ABCA1: ATP-binding cassette transporter A1
AcLDL: acetylated low-density lipoprotein
apoA-I: apolipoprotein A-I
CAD: coronary artery disease
DC-SIGN: dendritic cell-specific ICAM-3-grabbing nonintegrin
HAART: highly active antiretroviral therapy
HDL: high-density lipoprotein
LDL: low-density lipoprotein
LXR: liver X receptor
MHC: major histocompatibility complex
PI: protease inhibitor
RT: reverse transcription
SD: standard deviation
SR-B1: scavenger receptor B1
VSV: vesicular stomatitis virus
WT: wild type
ΔNef: Nef deficient
